# Psychological distress and associated factors among prisoners in North West Ethiopia: cross-sectional study

**DOI:** 10.1186/s13033-015-0033-7

**Published:** 2015-12-30

**Authors:** Berihun Assefa Dachew, Abel Fekadu, Teresa Kisi, Nigussie Yigzaw, Telake Azale Bisetegn

**Affiliations:** Department of Epidemiology and Biostatistics, College of Medicine and Health Sciences, University of Gondar, Gondar, Ethiopia; Department of Public Health, College of Medicine and Health Sciences, Arisi University, Assela, Ethiopia; Deaprtment of Psychiatry, College of Medicine and Health Sciences, University of Gondar, Gondar, Ethiopia; Department of Health Education and Behavioral Sciences, College of Medicine and Health Sciences, University of Gondar, Gondar, Ethiopia

**Keywords:** Ethiopia, Prisoners, Psychological distress

## Abstract

**Background:**

Currently, mental health is an important public health problem and the leading cause of disability worldwide. Studies have shown that, mental illnesses are more common among the prison population than the general population. However, still there is no accurate count of persons with mental disorder who are incarcerated in Ethiopia and information about prisoners’ health conditions is scarce. The purpose of this study was to assess the prevalence and associated factors of psychological distress among prisoner inmates found in prisons of Northwest, Ethiopia.

**Methods:**

Institution based cross sectional study was conducted among 649 prisoners from January to February 2015. Multistage sampling technique was used to select the study participants. Data were collected by using a structured interviewer administered questionnaire. Psychological distress was measured using the Kessler Psychological Distress Scale (K10). Receiver Operating Characteristic curve (ROC curve) analysis was done by STATA version 12 software in order to determine a cutoff point with high sensitivity and specificity. Bivariate and multivariable logistic regression models were fitted to identify associated factors. Adjusted odds ratio with its 95 % Confidence interval was used to declare the statistical significance between psychological distress and associated factors.

**Results:**

Prevalence of psychological distress among prisoners was found to be 83.4 % (95 % CI 80.6, 86.0 %). Long duration of stay in the prison (AOR = 0.95; 95 % CI 0.89–0.97), low to no satisfaction with prison services (AOR = 3.01; 95 % CI 1.38–6.51), and place of prison were factors significantly associated with psychological distress among prisoners.

**Conclusion:**

The prevalence of psychological distress among prisoners was found to be very high. Due attention needs to be given in addressing the mental health needs of the prisoners.

## Background

An estimated 10 million people are in prisons worldwide and the majority of them lives in low- and middle-income countries [1]. There are 450 million people worldwide who suffer from mental morbidity [2]. Although mental health problems affect society as a whole, studies have shown that mental illness is more common among the prison population than the general population [3, 4]. This can be due to overcrowding, various forms of violence, lack of privacy, lack of meaningful activity, isolation from social networks, insecurity about future prospects (work, relationships, etc.), and inadequate health services, especially mental health services, in prisons [5–7].

A study among prisoners in England and Wales [8] showed that 63 % of prisoners had psychological distresses. Furthermore, 70 % of prisoners in Ghana [9], 63 % of prisoners in Zambia [10], 61.9 % of Ethiopia [11] and 57 % of prisoners in Nigeria [12] experienced psychological distress.

Criminal offenders with mental disorders who do not undergo adequate treatment may enter in a cycle of recidivism regarding both the mental disorders and the criminal offenses [7, 13].

While these facts remain about mental illness and their contribution to the global burden of diseases, the attention given to mental health is very low across the globe in general and for prisoners in particular. This is even more so in low-income countries and still there is no accurate count of persons with mental disorder who are incarcerated in Ethiopia and information about prisoners’ health conditions is scarce.

However, knowing the mental health needs of prisoners is crucial in order for prison systems to develop appropriate health care programs for this population. Imprisonment may constitute an opportunity to reach medically underserved populations and addressing mental health needs of prisoners will improve the health and quality of life of both prisoners with mental disorders and of the prison population as a whole [14].

It is therefore a question of ethical importance and public health priority to study the type and frequency of psychological distress in prison populations. Hence, this study aimed to determine the prevalence of psychological distress and associated factors among prisoners imprisoned in prisons of Amhara regional state. The finding will provide data on the magnitude of mental disorders among prisoners; which will add some input for the country and assist policy makers in their effort to reform mental health care of prisoners in Ethiopia.

## Methods

### Study setting and design

Institution based cross sectional study was conducted to determine the prevalence of psychological distress and its associated factors among prison inmates found in prisons of North West Amhara regional state from January to February 2015. Amhara regional state is one of the 11 regions found in Democratic Republic of Ethiopia. The region covers a total area of 20,650,420 square KM and a total population of 19,602,512. There are 30 prisons in the region while 10 are found in the North Western part. The numbers of prisoners found in 30 prisons in the region were 22,590 while 7564 prison inmates were imprisoned in the three randomly selected prisoners found in the Northwest of the region.

### Sample size determination and sampling procedure

All prisoners found in selected prisons of the North West Amhara were the study populations. Those prisoners who were seriously ill were excluded from the study. The sample size (n) was computed by single population proportion formula [n = [(Z_a/2_)^2^*P (1 − P)]/d^2^] by assuming 95 % confidence level of Z a/_2_ = 1.96, 5 % margin of error, taking proportion of psychological distress approximately 0.65 [11].

$$n = \frac{{1.96^{2} \,\,\left( {0.65 \,(1 - 0.65} \right)}}{{0.05^{2} }} = 345$$

With this assumption the sample size becomes 345. Considering the design effect of two the final sample size was estimated to be 700.

Multistage sampling technique was employed to select the study participants. Three prisons: Bahir Dar, Debre Tabor and Gondar prisons were randomly selected from 10 prisons found in the Northwest Amhara Regional state. The prisoners’ list in every prison was used as a sampling frame and study participants were selected by using systematic random sampling technique.

### Data collection and data quality control

Data were collected by using structured interviewer administered questionnaire having five parts. The first part contains socio-demographic characteristics of the prisoners. The second part of the questionnaire assesses prisoner’s psychological distress by using the Kessler Psychological Distress Scale (K10). The K10 scale involves 10 questions about emotional states, each with a five-level response scale.The instrument has been used in several countries and its validity was established for Africa [15].

ROC curve analysis was done by STATA version 12 software in order to determine a cutoff point with high sensitivity and specificity. An individual is distressed if he/she has a score above the cutoff value, which is 12. The internal consistency of the tool was checked by conducting reliability test (Cronbach’s Alpha: 0.92).

The third part of the questionnaire was used to assess behavioral factors, which includes history of substance use (like Alcohol use, Chat chewing, cigarette smoking, Shisha) of the prisoner. The fourth part of is on the prisoners level of social support. Multidimensional scale of perceived social support (MSPSS) tool consists of 12 items was used to measure the level of prisoner’s social support. The last part assesses prisoners` social, economic and environmental factors.

Structured and Pretested questionnaire was used to collect the data. Eight trained B.Sc. holder data collectors conducted the data collection process. Collected data were reviewed and checked for completeness before data entry and incomplete data were discarded.

### Data processing and analysis

Data were checked, coded and entered to Epi Info version 7 and imported to SPSS version 20 for further cleaning and conducting univariate analysis. Both descriptive and analytical, statistical procedures were utilized. Tables and figures were used to present the data. The cleaned data were exported to R version 3.2.0 to fit Bivariate and multivariable logistic regression model to identify associated factors. Adjusted odds ratio with its 95 % Confidence interval was used to declare the statistical significance between psychological distress and associated factors. The variables were entered in the multivariable model using the Backward Stepwise (Likelihood Ratio) regression method.

### Ethical consideration

Ethical clearance was obtained from the Institutional Review Board (IRB) of University of Gondar. Permission to conduct the research was obtained from the regional prison administration agency and respective prison offices. Written consent was obtained from prisoners after explaining the purpose of the study. To ensure confidentiality their name and other personal identifiers were not registered in the format. It was explained to the participants that the selection to the study is random and they have the right to not respond for questions that they are not comfortable with. Finally, the questionnaires were kept locked after data entry is completed.

## Results

### Socio demographic characteristics of the respondents

A total of 649 respondents participated in the study with a response rate of 92.7 %. The mean (Standard deviation) age of the respondents was 30.6 (±11.5) years. Nearly 90 % of the respondents were males. About two-third of the respondents were urban in residence and 47 % of the respondents were single in marital status. Regarding their educational status close to 15 % of the respondents are able to read and write. Among the respondents, 12.9 % of them had a family history of mental illness (Table 1).

**Table 1:** Socio demographic characteristics of prisoners in Northwest Amhara, 2015 (n = 649)

Socio demographic variables	Frequency (%)
Sex	
Male	583 (89.8)
Female	66 (10.2)
Residence	
Urban	434 (66.9)
Rural	215 (33.1)
Religion	
Orthodox	584 (90)
Others (Muslim, catholic & protestant)	65 (10)
Marital status	
Single	306 (47.1)
Married	228 (35.1)
Not live with their partners	115 (17.7)
Educational status	
Not read and write	108 (16.6)
Read and write	97 (14.9)
1–8 class complete	129 (19.9)
9–12 class complete	206 (31.9)
Certificate and above	109 (16.8)
Family history of mental illness	
Yes	84 (12.9)
No	565 (87.1)

### Prisoner related characteristics

The mean duration of stay in prison was 9.6 (±5.5) years and 21.3 % of them are life sentenced prisoners. Most (82.8 %) of the prisoners participated in religious practice while they are in prison. Around 87 % of the respondents replied that they were happy with their previous life until they become a prisoner. However, currently more than half of the respondents reported that they often feel guilty and 43.6 % of the respondents feel that they had been discriminated by their families, friends and significant others because of their imprisonment. More than 85 % of the respondents believed that, the year they were penalized is not comparable with the crime they committed (Table 2).

**Table 2:** Prisoner related characteristics among prisoners in Northwest Amhara, 2015 (n = 649)

Variables	Frequency (%)
Type of prisoner	
Life sentenced prisoner	138 (21.3)
Other than life Sentenced prisoner	511 (78.7)
Frequency of conduct religious practice	
Always	308 (47.5)
Sometimes	229 (35.3)
Never	112 (17.3)
Participate in income generating activities	
Yes	389 (59.9)
No	260 (40.1)
Having a job before imprisonment	
Yes	467 (72)
No	182 (28)
Was happy with life before imprisonment	
Yes	567 (87.4)
No	82 (12.6)
Have a friend in the prison	
Yes	407 (62.7)
No	242 (37.3)
Having been discriminated because of imprisonment	
Yes	283 (43.6)
No	365 (56.4)
How often you feel guilty	
Always	354 (54.5)
Sometimes	105 (16.2)
Never	190 (29.3)
Perceived magnitude of crime committed	
Serious	304 (46.8)
Medium	152 (23.4)
Mild	193 (29.7)
Did you believe on the crime you have committed	
Yes	267 (41.1)
No	313 (48.2)
I don’t have any idea	69 (10.6)
Appropriateness of duration penalized with your crime	
Yes	30 (4.6)
No	557 (85.8)
I don’t have any idea	62 (9.6)
Satisfaction with the care you obtain	
Satisfied	65 (10)
Low to medium satisfaction	584 (89.4)
No satisfaction	4 (0.6)
Social support	
Yes	420 (64.7)
No	229 (35.3)

### Prevalence of psychological distress

Prevalence of psychological distress among prisoners was found to be 83.4 % (95 % CI 80.6, 86.0 %). Higher prevalence was observed among males (84.4 %) than females (74.0 %)

### Factors associated with psychological distress

In the multivariate logistic regression analysis, Long duration of stay in the prison (AOR = 0.95; 95 % CI 0.89–0.97), low to no satisfaction with prison services (AOR = 3.01; 95 % CI 1.38–6.51), and place of prison (Debre-Tabor (AOR = 1.26 95 % CI 0.63, 2.47), Gondar (AOR = 0.38; 95 % CI 0.19, 0.71)) were factors significantly associated with psychological distress among prisoners (Table 3).

**Table 3:** Multivariable logistic regression analyses of factors associated with psychological distress among prisoners in Northwest Amhara, 2015 (n = 649)

Explanatory variables	Psychological distress		COR,_95 % CI_	AOR,_95 % CI_
	Yes (%)	No (%)		
Sex				
Male	492	91	1	
Female	49	17	0.53 (0.29, 0.99)	
Length of stay in the prison			0.94 (0.91, 0.97)	0.93 (0.89, 0.97)*
Had you been discriminated because of your imprisonment				
Yes	244	39	1	
No	297	69	0.69 (0.44,1.05)	
How often do you feel guilty				
Always	53	301	1	
Sometimes	91	14	1.14 (0.62, 2.23)	
I never think	149	41	1.14 (0.41, 1.00)	
Satisfaction with the prison service				
Very satisfied	48	17	1	1
Low/No satisfaction	493	91	1.92 (1.03, 3.42)	3.01 (1.38, 6.51)*
Previous psychiatric problem				
Yes	84	8	1	
No	457	100	0.43 (0.19, 0.87)	
Is there a possibility that would prevent you to resettle to the previous state				
Yes	188	25	1	
No	353	83	0.56 (0.34, 0.90)	
Social support				
Yes	201	28	0.59 (0.36, 0.93)	
No	340	80	1	
Place of the prison				
Bahir Dar	216	24	1	1
Debre Tabor	160	30	0.59 (0.33,1.05)	1.26 (0.63, 2.47)*
Gondar	165	54	0.34 (0.19, 0.56)	0.38 (0.19, 0.71)*

* Significance at *P* value <0.05 AIC: 451.35

## Discussion

In the current study the prevalence of psychological distress among prisoners was found to be 83.4 % (95 % CI 80.6, 86.0 %). High prevalence rates of psychological distress among prisoners were found in this study as compared to studies done in Ghana (70 %) [9], Zambia (63 %) [10], Nigeria (57 %) [12], England and Wales (63 %) [8], Brazil (39.2–68.9 %) [16], Iran (57.2 %) [17] and Switzerland (43.5 %) [18]. This discrepancy may be due to the difference in the sampled populations, socio cultural and environmental factors. It may also due to the difference in the service provided in the prison. For example, in this study only few (10 %) of the prisoners are satisfied with the service they received which may lead them to be stressed. On the other hand, majority (87 %) of the prisoners felt happy with their life before they become prisoner. However, Similar findings were observed in a study carried out in the USA, where 66 % to 85 % of the prisoners had mental illness [19].

The current study prevalence of psychological distress is higher among males (84.4 %) than females (74.0 %). This finding is contrary to other studies done so far [10, 15, 16, 20]. This can be due to the sample size difference i.e. the sample of females is too small (n = 66) in this study as compared to males (n = 583).

In the current study duration of stay in the prison is significantly associated with psychological distress. A unit increase in length of stay in the prison results in a decrement of psychological distress by 0.95 (β = 0.95; 95 % CI 0.89–0.97). It is to mean that as a prisoner stayed longer in the prison s/he will approach to her/his release and her/his stress will go down. Since the prison environment is more stressful than the normal institutional life due to overcrowding, lack of privacy, lack of meaningful activity, various forms of violence, isolation from social networks, and inadequate mental health services in prisons, anticipating their release from such an environment will reduce the prisoner’s stress level.

The study also found that psychological distress was significantly associated with prison service satisfaction. The odds of having psychological distress were three times higher among prisoners who were not satisfied with prison services as compared to those who were satisfied. Moreover, this study revealed that type of prison is significantly associated with psychological distress. The odds of psychological distress were 1.26 times higher for prisoners who were imprisoned in Bahir-Dar than in Debere-tabor. On the other hand, psychological distress is reduced by 62 % among prisoner in Gondar as compared to Bahirdar. This can be attributed to the difference in prison settings and the differences in services available in the prison.

This study has some limitations that can be considered while interpreting the results. Since the data were collected by interview method and the interviews were performed in prisons the study may be prone to social durability bias. In addition, the reports for some of the questions were past history or encounters which are prone to recall bias. Moreover, we used Kessler-10 tool to assess psychological distress. Since the tool only assesses broad, non-specific psychological distress, rather than specific mental disorders, higher psychological distress scores observed in this particular prison does not imply estimates of clinical need among the prisoners. Furthermore, prisoners more likely to exaggerate psychological distress that could have overestimated the prevalence.

## Conclusion and recommendation

The prevalence of psychological distress among prisoners was found to be very high, indicating prisoners are highly vulnerable to mental illness as compared to the general population. Addressing mental health needs of the prisoners will not only benefit the prisoners, but also the prison employees and the larger community too. Therefore, due attention should be given in addressing the mental health needs of the prisoners.

Addressing the needs of people with mental disorders improves the probability that upon leaving prison, they will be able to adjust to community life, which may, in turn, reduce the likelihood that they will return to prison. Moreover, it decreases incidents of re-offending and reduces the number of people who return to prison. In addition, it will also help to divert people with mental disorders away from prison and ultimately reduce the high costs of prisons. We recommend nationwide more comprehensive research to determine the true prevalence and determinant factors of psychological distress among all incarcerated individuals in Ethiopian.

## Abbreviations

AOR: Adjusted odds ratio
IRB: Institutional Review Board
K10: Kessler Psychological Distress Scale
ROC: Receiver operating characteristic curve
SPSS: Statistical package for social sciences
